# An analysis of alternative forced oscillation technique reporting and validation methods for within- and between-sessions in healthy adults

**DOI:** 10.1038/s41598-022-17264-2

**Published:** 2022-07-30

**Authors:** Jennifer H. Therkorn, Wei Qian, Daniella R. Toto, Michael J. Falvo

**Affiliations:** 1 Airborne Hazards and Burn Pits Center of Excellence, War Related Illness and Injury Study Center, VA New Jersey Health Care System, 385 Tremont Ave, East Orange, NJ 07018 USA; 2 Rutgers New Jersey Medical School, Rutgers Biomedical and Health Sciences, Newark, NJ USA; 3 School of Osteopathic Medicine, Rowan University, Stratford, NJ USA; 4 Department of Pharmacology, Physiology and Neuroscience, Rutgers New Jersey Medical School, Newark, NJ USA; 5 Department of Physical Medicine and Rehabilitation, Rutgers New Jersey Medical School, Newark, NJ USA

**Keywords:** Respiration, Medical research

## Abstract

Forced oscillation technique (FOT) provides unique information on respiratory system mechanical properties complementing pulmonary function testing. However, a lack of evidence guiding acquisition/reporting of parameters has slowed clinical FOT adoption. Current European Respiratory Society (ERS) standards recommend 3–5 trials per session comprising three trials with a coefficient of variation (CoV) ≤ 10% for low-frequency resistance. We present an analysis of different combinations of trial selection methods and session validity thresholding variables (low- and mid-frequency resistance and reactance [R5, R19, X5], low-frequency reactance area [AX] and tidal volume) comparing proportion of subjects achieving valid data across two test sessions (7 ± 3 days apart) and within and between session measurement variabilities. 126 (98%) subjects achieved valid data across both sessions (2666 trials). With R5 or R19 as criteria and selection of any three trials from ≥ 4 attempts, ≥ 75% of subjects achieved validity. Furthermore, with R5 or R19 criteria and selection of any trials from ≥ 5 attempts, CoVs for resistance outcomes were reduced within session while variabilities of FOT outcomes between sessions remained consistent. Within session differences in measurement variabilities were not clinically meaningful. Our analyses support current ERS reporting recommendations for healthy adults. Future work should apply this analytic approach to patient populations.

## Introduction

Clinical adoption of the forced oscillation technique (FOT), or oscillometry, has been gradual despite offering unique information about respiratory system mechanical properties obtained through quiet tidal breathing. There are several factors that contribute to the protracted uptake of FOT including, but not limited to the following: (1) lack of standardization across commercially available devices^1^, (2) limited reference values from diverse populations^2^, (3) inconsistency in testing protocols and reporting^3^, and (4) an absence of data to inform test selection methods^3^. To address these knowledge gaps, there has been significant efforts underway amongst many laboratories as well as the recent Technical Standard report from the European Respiratory Society (ERS;^3^) to improve standardization.

The recent ERS Technical Standard underscored the need for increased transparency, quality control and consistency across device (and software) manufacturers^3^. Harmonization efforts amongst manufacturers is underway and are expected to evolve^1^. Independent of these efforts, however, remain fundamental questions about FOT measurement acquisition and reporting. For adults, current ERS recommendations are that the FOT indices of resistance and reactance be derived from the mean of three artifact-free trials or measurement replicates of which the lowest frequency resistance have a coefficient of variation (CoV) ≤ 10%. However, if more than three replicates are performed (which is common), it is not currently well described which exact three measurement replicates within a testing session are to be used to derive the mean indices; furthermore, there has been no quantitative evaluation of other potential criteria which may serve as the CoV thresholding variable (e.g., low versus mid-frequency, resistance versus reactance).

Harkness et al.^4^ recently addressed the lack of data on CoV thresholds and measurement selection for different adult populations (healthy, asthma or chronic obstructive pulmonary disease [n = 15 per group]). In brief, they obtained eight measurement replicates in all individuals and evaluated a range of target CoV thresholds (i.e., ≤ 5% to 20%) using a combination of selection methods for repeat trials (i.e., all eight, first three, and closest three measurements) considering low-frequency resistance (5 Hz). Applying the current ERS within-session variability criteria, Harkness et al. found most healthy adults could achieve this criterion within four measurements whereas those with lung disease needed up to six measurements. Moreover, when selecting the ‘closest’ three of eight measurements for resistance at 5 Hz, they observed CoV’s that were less than 3% for all individuals.

The aforementioned study^4^ provides the field important information regarding the effects of manipulating the current ERS criteria for target CoV cut-offs as well as different combinations of trial selection methods. However, key aspects of the process used to select which trials represent a given test session and how these processes impact variability of FOT indices are not fully addressed by any previous research. Specifically, these aspects include a) the joint effect of varying both trial selection method and which FOT metric is used as the within session variability thresholding criteria, and b) the impact of these different selection method/criteria combinations on both within and between session variability. In the present study, we address these aspects using a two phased systematic analysis of alternatives by comparing the performance of different selection method/criteria combinations versus what we term here as the current ERS status quo (i.e., three closest measurements with a low-frequency resistance CoV ≤ 10%)^3^. The two phases of the analysis include (1) comparison of the proportion of subjects able to achieve valid data across two test sessions (basis of practicality) and (2) reduction of within and between session measurement variability (basis of test quality). Overall, we present methodology and results that can inform a rationale for FOT trial reporting and support future standardization.

## Results

### Participants

Complete data were available for 94% of our sample (n = 129). A detailed description of this sample has previously been reported^5^, including reasons for missing data as well as lifestyle and behavior characteristics. The majority of our participants were female (72%, 93/129), white (44%, 57/129) and non-Hispanic or non-Latino (85%, 110/129). Self-reported sleep quality and mood were similar between sessions. On a 0–100 visual analog scale with 100 indicating ‘very comfortable’ and ‘very easy’, participants rated FOT as being easy (Session 1 and Session 2; mean ± SD: 86.5 ± 19.2, 88.3 ± 17.7) and comfortable (91.8 ± 12.8, 91.2 ± 14.9) to perform.

### Session and trial characteristics

Median between-visit duration was 6.1 days (lower quartile, upper quartile [IQR]: 6.0, 7.0). Across both test sessions, a total of 2666 FOT trials were attempted by participants, of which 78% (2082/2666) were valid following software and investigator quality checks (Table 1). Of the 574 trials rejected, 97% of these (556/574) were due to automated software rejection and 3% (18/574) were due to investigator inspection. While 129 subjects participated in both sessions, only 126 (98%, 126/129) of these subjects were able to achieve valid data post quality checks in both sessions. At the individual subject level, 13% to 100% of all trials attempted within a subject’s session passed quality checks with a mean number of valid trials per subject of eight (range: 1 to 20).

**Table 1:** Trial characteristics across study sessions.

	Session 1	Session 2	Combined
**Trials summary**			
Attempted trials, n	1356	1310	2666
Trials rejected by software, n (%)	309 (23%, 309/1356)	247 (19%, 247/1310)	556 (21%, 556/2666)
Trials rejected by investigator, n (%)	11 (< 1%, 11/1356)	7 (< 1%, 7/1310)	18 (< 1%, 18/2666)
Valid trials post software and investigator inspection, n (%)	1036 (76%, 1036/1356)	1046 (80%, 1046/1310)	2082 (78%, 2082/2666)
**Subjects summary**			
Subjects participating post exclusion criteria, n	133	129	129
Subjects with valid data post software and investigator inspection, n (%)	131 (98%, 131/133)	127 (98%, 127/129)	126 (98%, 126/129)
Valid trials per subject, mean ± SD (range)	7.9 ± 2.5 (1, 20)	8.3 ± 2.5 (1, 19)	8.1 ± 2.5 (1, 20)
Percent (%) valid trials per subject, median (range)	85% (13%, 100%)	90% (25%, 100%)	90% (13%, 100%)

*SD* 1 standard deviation.

### Analysis phase 1: down selection of criteria and trial selection method combinations

**Figure 1:**
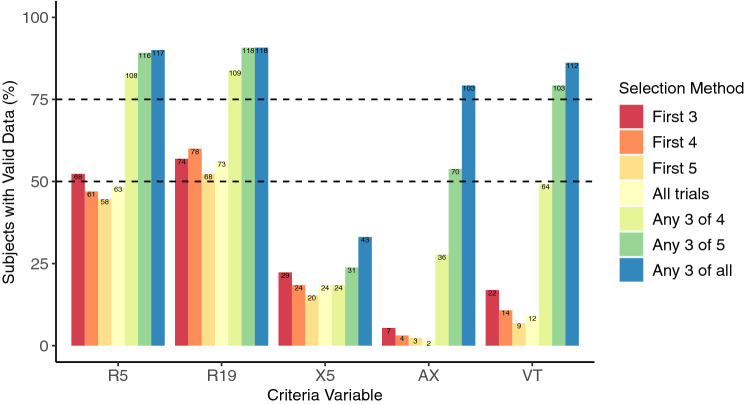
Proportion of subjects with valid data as a function of selection method. Percent (y-axis) and number (top of bars) of subjects able to achieve valid data for each selection method and criteria combination across both sessions. The percentages are out of a total of 129 (number of subjects attempting trials in both sessions). Valid data refers to trials passing three levels of quality assurance: (**1**) software checks, (**2**) investigator checks, and (**3**) session validity following trial selection and application of the thresholding criteria variable (CoV ≤ 10%). Reference lines (black dashed lines) are drawn at 50% and 75%.

The goal for the first phase of analysis was to investigate the proportion of subjects able to achieve valid data for each selection method/criteria variable combination as a measure of practicality for larger population-based studies. Generally, as flexibility increased to choose any combination of trials from ≥ 4 attempts to obtain the lowest criteria variable CoV, the number of subjects able to achieve valid data increased (Fig. 1). In Fig. 1, reference lines illustrate for which selection methods ≥ 50% and ≥ 75% of subjects were able to achieve a valid test session. These lines are arbitrary, yet they guide reporting of results and discussion. When using low-frequency reactance at 5 Hz (X5) as the criteria, none of the selection methods resulted in achieving either benchmark. When using either reactance area (AX) or tidal volume (V_T_) as criteria, both benchmarks could be achieved but only if ≥ 5 trials (and any combination thereof) were considered. When using both resistance at 5 and 19 Hz (R5 and R19) as the criteria, this resulted in multiple different selection methods surpassing benchmarks where fewer than five trials were considered; for both of these criteria (R5 and R19), this included the method ‘first 3’ representing the first possible sequence of trials attempted which could be considered a complete test session. However, both criteria required flexible choice of any combination of three trials from ≥ 4 attempted to surpass the 75% benchmark. For these reasons, the following selection methods were down selected to proceed to the next phase of analysis considering only R5 and R19 as criteria: ‘first 3’, ‘any 3 of 4’, and ‘any 3 of 5’. It should be noted, however, that consideration of > 5 attempts with a resistance based threshold criteria did not result in an increased number of subjects able to achieve session validity. Generally, any subjects achieving session validity did so within five attempts when selecting any combination of three trials giving the lowest resistance-based CoV. Using R5 or R19 as the criteria, the number of subjects able to achieve valid data across all selection methods progressed to Phase II and within both sessions were 68 and 74, respectively.

### Analysis phase 2: evaluating criteria/selection method impact on within and between session measurement variability

**Figure 2:**
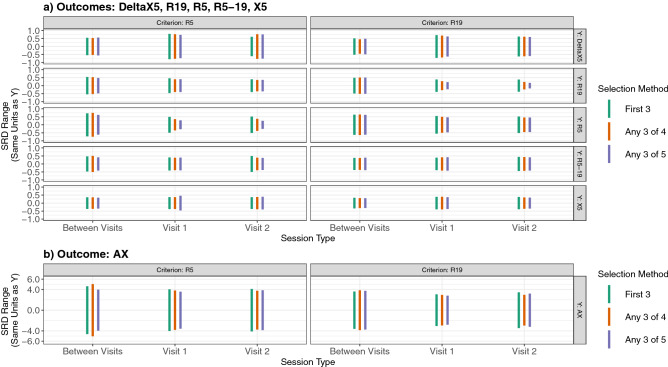
Minimum smallest real difference (SRD) range within and between sessions for each trial selection method stratified into two vertical sets of panels by coefficient of variation thresholding criteria and six horizontal sets of panels by outcome used as model fit response (Y’s). The horizontal sets of panels stratified by outcome were split into sections “a” and “b” to account for the different y-axis scale associated with reactance area, AX. SRD units are the same as the outcomes, so the y-axis scales vary by outcome metric as follows. In panel “a”: outcomes (low- (5 Hz) and mid-frequency (19 Hz) resistance (R5, R19) and reactance (X5), frequency dependence of resistance (R5-19), delta X5 (∆ = X5 inspiratory—X5 expiratory;^14^), cm H_2_O ·s/L). In panel “b”: reactance area (AX, cm H_2_O/L). SRD was calculated as either the within or between session standard deviation (i.e., root mean square error (RMSE) from model fits) multiplied by √2 × 1.96 (SRD = RMSE × 2.77)^25^. What is termed here as the minimum SRD range was calculated as zero (i.e., assuming no difference between measurements using the same approach) ± SRD. Therefore, smaller minimum SRD ranges represent less measurement variability and greater sensitivity to detect a true difference^24,25^.

The overall goal for the second phase of analysis was to identify an optimized choice for criteria and selection method defined as one that minimized contributions to measurement variability while maintaining true variability; we assessed this using the minimum smallest real difference (SRD) range. Across all FOT outcome measures explored, which criteria and selection method combination was used to drive the validity determination of the test session appears to have had limited impact on how test session results would be interpreted clinically. Qualitatively, this especially appears to be the case for between visit variabilities as illustrated by the similarity in minimum SRD range lines (Fig. 2). Figure 2 demonstrates that the only case under which a difference in minimum SRD ranges existed was when the variable used for within session CoV thresholding was the same as that for which outcome variability was being assessed (i.e., R5 and R19). But even under these conditions, the SRD ranges across selection methods were found to be at most 0.26 to 0.52 units different (cm H_2_O ·s/L) when comparing methods ‘first 3’ vs. ‘any 3 of 5’.

Because there were no clinically meaningful differences in variabilities obtained using either R5 or R19 as the thresholding criteria (Fig. 2), Table 2 presents a summary of model fit results across selection methods for all outcomes using R5 (i.e., the status quo criteria). Within each test session, all selection methods resulted in model fit CoV’s < 6% for both R5 and R19 outcomes. R5 and R19 CoV tended to decrease as flexibility increased in selecting from a greater number of trials giving the lowest R5 CoV. However, having more trials to choose from did not reduce CoV when comparing between sessions; all selection methods resulted in similar CoV’s for R5 and R19 between sessions ranging from about 6–7%. Variability within and between sessions for all other outcomes (R5-19, X5, Delta X5, AX) as illustrated by SRD% and/or CoV tended to be much greater than that achieved for either R5 or R19 with no apparent trends in variability versus selection method. There was, however, relatively consistent variability observed for these outcomes in comparing the results obtained within versus between test sessions.

**Table 2:** Within and between session variability across model fit response variables (Y’s) for each trial selection method using R5 as the session validity thresholding criteria.

Y	Variability metric	Within session 1			Within session 2			Between sessions		
		First 3	Any 3 of 4	Any 3 of 5	First 3	Any 3 of 4	Any 3 of 5	First 3	Any 3 of 4	Any 3 of 5
R5	RMSE	0.18	0.13	0.10	0.18	0.13	0.09	0.26	0.27	0.22
	SRD	0.49	0.35	0.27	0.51	0.37	0.25	0.71	0.74	0.61
	SRD%	14.43	10.25	8.03	15.21	11.22	7.48	21.23	22.00	18.19
	CoV	5.21	3.70	2.90	5.49	4.05	2.70	7.66	7.94	6.57
R19	RMSE	0.17	0.14	0.14	0.14	0.13	0.13	0.20	0.19	0.17
	SRD	0.46	0.39	0.40	0.40	0.35	0.36	0.54	0.53	0.48
	SRD%	15.44	13.23	13.36	13.48	12.01	12.12	18.23	17.75	16.13
	CoV	5.57	4.78	4.82	4.87	4.34	4.37	6.58	6.41	5.82
R5-19*	RMSE	0.15	0.14	0.14	0.18	0.14	0.14	0.17	0.18	0.15
	SRD	0.41	0.39	0.40	0.50	0.39	0.38	0.47	0.50	0.41
	SRD%	102.07	97.29	99.29	130.75	102.94	98.55	120.41	128.47	106.15
X5	RMSE	0.13	0.13	0.17	0.13	0.14	0.14	0.13	0.13	0.12
	SRD	0.37	0.37	0.46	0.37	0.38	0.39	0.36	0.36	0.34
	SRD%	30.57	30.28	37.73	31.25	32.12	33.26	30.27	29.89	28.32
	CoV	11.04	10.93	13.62	11.28	11.60	12.01	10.93	10.79	10.22
Delta X5*	RMSE	0.29	0.28	0.26	0.22	0.28	0.27	0.20	0.19	0.20
	SRD	0.79	0.76	0.72	0.61	0.77	0.76	0.54	0.53	0.56
	SRD%	211.15	204.31	193.71	179.79	227.77	224.10	153.08	148.30	157.04
AX	RMSE	1.45	1.38	1.29	1.48	1.35	1.39	1.67	1.82	1.43
	SRD	4.03	3.81	3.57	4.11	3.75	3.85	4.63	5.04	3.97
	SRD%	65.18	61.65	57.82	67.23	61.36	63.01	75.32	82.02	64.55
	CoV	23.53	22.26	20.87	24.27	22.15	22.75	27.19	29.61	23.30

R5, R19 and X5 = low- (5 Hz) and mid-frequency (19 Hz) resistance and reactance, respectively; R5-19 = frequency dependence of resistance; delta X5 = X5 inspiratory—X5 expiratory^14^ [all in units of cm H_2_O ·s/L]; AX = reactance area [cm H_2_O/L]; RMSE = standard deviation from model fit (root mean square error); SRD = smallest real difference = RMSE × 2.7^25^; SRD% = SRD/(mean of response) × 100; CoV = RMSE/(mean of response) × 100.

* = variable spanning positive and negative values, so CoV is not calculated. Summary data for each variable, including response means used in calculations, can be found in the Supplementary Materials (Figure S1, Table S1).

## Discussion

The present study sought to systematically identify which combination of criteria and selection methods for the calculation of FOT-derived indices yielded the least within- and between-session variability while being practical to perform. Motivating this work, in part, was the recent ERS Technical Standard report^3^ that highlighted the paucity of published data to support current acquisition and reporting recommendations—i.e., mean of three measurements with a low-frequency resistance CoV ≤ 10%. Though we included the current ERS status quo standard in our analysis, we broadened our approach to consider alternative parameters and measurement numbers. Using a two-phase analytical approach, we first considered the proportion of subjects able to achieve valid data followed by analysis of measurement variability (within- and between-session). Irrespective of criteria variable (R5, R19, X5, AX, V_T_), selecting any three measurements from all available valid measurements yielded the largest proportion of participants having a complete test; although, R5 and R19 used as the criteria variable resulted in the largest proportion of subjects with valid data and subjects were generally able to achieve validity within five trials. We subsequently applied these criteria (R5, R19) to evaluate selection method variability within- and between-sessions.

Considering only subtle differences were found across FOT outcome variabilities obtained between different selection method/criteria combinations, the key finding of the present study is that there appears to be no evidence refuting continued use of the current ERS status quo trial selection and reporting method for healthy adults, i.e., method ‘any 3 of 5’ using R5 as the session validity criteria variable. How the field arrived at 3–5 trials is not readily apparent as early descriptions of FOT described reporting the mean of “at least eight individual measurements (p. 2047,^6^)” or “five separate 10-s periods (p. 1214,^7^)”. Nevertheless, it does appear that the current rationale for FOT reporting is theoretically driven and based on several factors. First, R5 is thought to better represent the small airways as the lower frequencies (i.e., 4–6 Hz) are able to travel deepest into the lungs. Second, relative to reactance, resistance should be independent of frequency in healthy adults. Third, the use of whole breath R5 considers resistance measured during both inspiration and expiration. Many devices do not allow for partitioning between the two. Therefore, low frequency, whole breath resistance can be implemented as the criteria regardless of FOT device used to acquire the data. Given the theoretical, historical and practical basis for using R5 as the criteria and our two-stage analysis of alternatives results presented here, we recommend continued use of the current ERS status quo for healthy adults.

Generally, the results obtained in the present study for within session variability for R5, R20, and X5 are comparable to similar previous studies implementing the same selection methods with R5 as the criteria for healthy subjects^4,8^. Previous studies, however, did not explore how various selection methods impact between session variability. Our results suggest that the benefit of reduced variability when considering a greater number of trials to select from does not extend across different test sessions. For example, with R5 as criteria, selection methods ‘first 3’, ‘any 3 of 4’, and ‘any 3 of 5’ resulted in R5 model fit outcome CoV’s of about 5%, 4%, and 3%, respectively, within sessions. Between sessions, these methods yielded CoV’s of about 8%, 8%, and 7%, respectively. This outcome likely reflects that selection methods are only applied at the within session level with no quality assurance criteria imposed across different sessions.

### Strengths

As recently described by Kouri et al.^9^, the development and refinement of FOT parallels the evolution of spirometry with respect to technological advancements as well as the standardization of technique and variable reporting. Spirometry, however, was first introduced more than 110 years before the first FOT device was described by DuBois et al.^10^. Therefore, the present study, and the critical work that predates our efforts, are fundamental to advancing implementation of FOT as a modality. Our approach has several key strengths in this regard. Foremost, we designed this study to ensure data were acquired in a rigorous and systematic manner as detailed in our methods section. Key aspects include standardized verbal and visual instruction, single technician for acquisition and protocolized quality review. Our sample was large (n = 126 for final analyses) relative to related work in this area and included two sessions that afforded assessment of both within- and between-session variability^4,11^. Additionally, this study adds new knowledge with respect to consideration of multiple selection methods and criteria as well as a broad array of frequently reported FOT parameters (R5, R19, R5-R19, X5, deltaX5, and AX). This comprehensive strategy is enhanced by a thorough analytical approach and transparent reporting in the main manuscript as well as Supplementary Material.

### Limitations and future directions

FOT data were acquired on a single commercially available device that is widely used. Given device-specific differences on signal processing and other instrument factors^1^, our results should be interpreted with some caution. However, the systematic approach and nature of our work has application beyond a single device. We also purposefully delimited our sample to healthy non-smoking young adults (18–40 years) and therefore cannot assume that our findings are robust to other populations and/or older adults. From a technical perspective, the recent ERS Technical Standard^3^ recommends a 30 s measurement recording time for adults to ensure acquisition of at least three artifact free breaths. It is recognized that this duration varies based on the population being tested, and since we enrolled healthy young adults, we selected a 20 s recording time to achieve ≥ 3 artifact free breaths. Watts et al.^12^ compared measurement recording times (16, 30, and 60 s) in healthy adults and those with asthma and COPD and found no clinically relevant differences provided that ≥ 3 acceptable breaths were achieved. In the present study, we set a minimum threshold of ≥ 3 breaths but did not analyze the effect of breath number across measurement replicates nor final variable reporting. Future studies may consider this type of analysis between respiratory cycles in light of growing attention to intra-breath oscillometry^13^.

While criteria R5 allowed for decreasing within session CoV for R5 outcomes (and potentially to some extent for R19), using a resistance-based criterion did not have any apparent benefits in reducing variability for the other outcomes (Fig. 2). The impacts on clinical interpretation of these other outcomes and future studies should be considered. For example, studies have shown potential utility in Delta X5 for detecting expiratory flow limitation in patients with chronic obstructive pulmonary disease^14^. Delta X5 SRD% values were calculated ranging from about 150–225% (Table 3). In other words, the SRD for Delta X5 was found to be about 1.5 to 2× its mean value suggesting a high degree of measurement variability. Given this high degree of variability, future work focused on FOT outcomes such as Delta X5 may have difficulty in detecting true differences in these variables. Exploration of the more highly variable yet less common FOT outcomes as potential criteria variables was outside the scope of the present study. Perhaps, optimization of selection methods/criteria combinations may need to be tailored to whichever FOT outcomes are of interest for a given study, but the impacts of these approaches on cross study comparability using different methods must also be considered.

**Table 3:** Methods investigated for within session trial selection.

Selection method	Number of trials selected	Method description
First 3	3	First 3 valid trials selected in sequential order
First 4	4	First 4 valid trials selected in sequential order
First 5	5	First 5 valid trials selected in sequential order
All	All	All valid trials selected
Any 3 of 4	3	Any 3 trials providing lowest CoV for criteria variable from first 4 valid attempts
Any 3 of 5*	3	Any 3 trials providing lowest CoV for criteria variable from first 5 valid attempts
Any 3 of all	3	Any 3 trials providing lowest CoV for criteria variable from all valid attempts

*CoV* Coefficient of variation (%).

*Current ERS standard (status quo).

### Conclusion

FOT offers unique information about the respiratory system that complements other pulmonary function testing modalities and has seen rapid growth in the scientific literature. Widespread clinical adoption of FOT, however, has been slow for a variety of reasons including, but not limited to, a lack of data regarding how to acquire and report measurements. The present study addresses the latter concern in healthy adults using a rigorous and transparent approach. Fortunately, our analyses suggest that clinicians and researchers should have confidence in applying the current ERS standard for FOT acquisition and reporting (i.e., method ‘any 3 of 5’ with R5 as criteria) such that within- and between-sessions variability is minimized yet practical to perform. Replication of our findings using other commercially available FOT devices as well as for different age groups and clinical conditions is necessary and would provide additional assurance to the field.

## Methods

### Participants

Participants (n = 137) between the ages of 18 to 40 years were recruited from the regional area, and screened based on medical history, smoking history and body composition. Individuals were excluded if they met any of the following exclusion criteria: any contraindication to spirometry or current medication use that can affect lung function^15^, e-cigarette use or vaping ≥ once per month or use of traditional tobacco products within the last 12 months (former smokers with ≤ 5 lifetime pack years could be included), any major organ disease or cancer, childhood asthma, pregnant or morbid obesity (≥ 40 kg/m^2^). Participants were instructed to refrain from vigorous activity 24 h prior to their visit and had to deny respiratory illness or infection within the prior three weeks. All participants provided their written informed consent and procedures were approved by the Rutgers University Institutional Review Board. All research was performed in accordance with relevant guidelines and regulations, including all ethical principles for research involving human subjects.

### Experimental design

As part of a larger study^5^, participants attended two separate laboratory sessions one week apart (± 3 days) at the same time of the day (± 2 h). During the first visit, demographic and anthropometric information were obtained, including their height, weight and waist-to-hip measurement^16^. Participants self-reported their prior knowledge or experience of breathing tests, comfort and ease with FOT, level of physical activity, quality of sleep and mood state using REDCap electronic data capture tools hosted at Rutgers University^17,18^. Details regarding these questionnaires have been previously reported^5^. Following completion of questionnaires, participants were provided instructions and then performed FOT followed by spirometry. Spirometry results have been previously reported and are not discussed herein^5^.

### FOT protocol

Prior to commencing testing, an investigator read aloud to each participant a standardized script that included the recommended minimum instructions (c.f., Table 5^3^) as well as a visual demonstration. FOT was performed using a commercially available device (TremoFlo C-100, Thorasys Medical Systems; Montreal, Canada) that employed a pseudo-random oscillation signal within the 5–37 Hz (relative primes) frequency range. Data were acquired using the manufacturer’s software (version 1.0.43) and the device was physically secured to a table using a variable friction arm with clamp (Manfrotto 244 N, 035; Ramsey, New Jersey). The variable friction arm allowed the technician to achieve the correct head position for each subject. Participants supported their own cheeks in accordance with verbal and visual guidance and donned a nose clip during testing. Emphasis was placed on proper posture with elbows slightly flared and maintaining a firm seal around the anti-bacterial filter. A more detailed description of this protocol, including a video, is available elsewhere^19^.

A verification procedure was performed prior to measurement acquisition according to manufacturer specifications using a calibrated test load device of known resistance. A single trained technician acquired at least 10 consecutive measurements (20 s recording epoch), interspersed with 30–60 s of rest between replicates. Valid measurements were defined as meeting the following criteria: ≥ 3 whole breaths and ≥ 70% valid data after outlier removal (described under ‘Data Quality and Reduction’), inspiratory and expiratory times ≥ 0.35 s, and tidal volume (V_T_) ≥ 100 mL. Impedance values were derived from the mean for each measurement for whole breaths. Our primary variables of interest included low- (5 Hz) and mid-frequency (19 Hz) resistance (R5, R19) and reactance (X5), frequency dependence of resistance (R5-19), low-frequency reactance area (AX), delta X5 (∆ = X5 inspiratory—X5 expiratory;^14^), and V_T_.

### Data quality and reduction

The manufacturer’s software-based outlier detection algorithm was employed to identify artifacts. This algorithm rejects data points within a measurement period if the resistance is ≥ 3 standard deviations from the mean or if the resistance is negative. The following thresholds were used to flag data for manual inspection: V_T_ > 2 L, R5 > 6 cmH_2_O·s/L, or X5 < -3 cmH_2_O·s/L. All flagged trials were individually inspected by a single investigator to assess for potential artifacts missed by the software or other potential indicators of suboptimal data (i.e., low coherence or unusual tracings). Each excluded trial was recorded by the investigator with rationale in a spreadsheet.

### Analysis phase 1: down selection of fot criteria and trial selection methods to test

An analysis of alternatives methodology was employed to compare different combinations of criteria and trial selection methods versus the status quo ERS method. In this first phase of analysis, the proportion of subjects able to achieve valid data across two different study sessions for each combination was explored. This phase was focused on practicality; an overly restrictive method that cannot be achieved by the majority of healthy participants in a study would be impractical to implement. The objective of this phase was down selection to a smaller subset of criteria/selection methods to proceed to the second phase of analysis.

Seven different methods were compared for selecting trials within a session under the requirement that a complete test session should comprise ≥ 3 artifact free trials (Table 3). Each of these selection methods were performed after data quality and reduction procedures described above; therefore, only valid trials were considered for selection. The selection methods explored have all been previously described in past studies and are simple to implement^3,4^. All methods investigated were based on the selection of multiple trials to enable statistical model fitting as opposed to, for example, methods reliant on selection of a single trial to represent test sessions for large epidemiological studies^8^. Finally, all selection methods employed a criteria threshold for inclusion wherein the selected trials had to provide a CoV ≤ 10%^3^. The following FOT indices were tested as the criteria variable: R5, R19, X5, AX and V_T_. These variables represent FOT outcomes that span exclusively either positive or negative values therefore allowing for CoV calculation. While V_T_ is not FOT specific per se, it has been recommended to ensure oscillometric measurements are performed during a period of stable tidal breathing without explicit definition to what this means^3^. After selecting trials within each test session, the mean was reported across all outcomes for between session analysis.

### Analysis phase 2: evaluating criteria/selection method impact on within and between session measurement variability

In the second phase, an analysis approach based on random effect or mixed effects models was used to assess differences in measurement variability within and between sessions. This method has been similarly used in previous research for evaluating within and between session variability for spirometry selection methods^5^. For the purposes of the present study, an optimized choice for criteria and selection method was considered one that minimized contributions to measurement variability while maintaining true variability. To achieve the objectives for this phase of analysis, within and between session variability attributable to criteria and selection methods were estimated by deriving the common within subject standard deviation (i.e., root mean square error, RMSE)^20^ from random effect models fit post implementation of trial selection using different selection methods and criteria variables. These random effect models included only one predictor variable as a random effect (subject) and investigated relative differences between criteria/selection method combinations on measurement variability for these FOT outcomes: R5, R19, R5-19 difference, X5, delta X5, and AX. For the between session analyses, visit number was also included as a fixed effect (i.e., mixed effects models).

Model diagnostics, including Pearson residuals vs. fit, QQ plots for random effects, and predicted fit vs. observed data, were checked to ensure model assumptions were satisfactory. Diagnostic plots indicated deviation from model assumptions when the outcome variable was skewed, particularly the assumption of normality of random effects. (Histograms illustrating outcome variable distributions can be found in the Supplementary Materials (Figure S1)). Therefore, outcome variables were transformed as follows – natural log transformation was performed for R5 and AX; X5 was multiplied by -1 and the natural log taken^21^; a constant of + 1 was added to R5-19 to shift the distribution to all positive values and then natural log was taken. As identified using the afex package in R, the bobyqa control optimizer was used to facilitate model convergence^22^. Because the only value of interest from each model fit was the RMSE, sensitivity checks were performed for potential influences on RMSE caused by influential data points as identified by Cooks distance using the influence.ME package in R^23^. Additionally, sensitivity analyses were performed using two different subject datasets: (1) all available data: any subjects able to achieve valid data for each criteria/selection method combination in either study visit, and (2) conservative data: only subjects who could achieve valid data across all criteria/selection method combinations and in both sessions. The conservative analyses are presented in the manuscript, while the analyses performed on all available data are presented in the Supplementary Materials (Figure S2, Table S2).

RMSE was reported as either the within session or between session standard deviations based on model type. CoV was calculated as the ratio of RMSE to mean of outcome variable within or between sessions (CoV = RMSE/(mean of response) × 100)^20^. The repeatability or smallest real difference was calculated as RMSE multiplied by √2 × 1.96 or 2.77 (SRD; SRD = RMSE × 2.77)^24^, and SRD% was calculated as ratio of SRD to mean of response either within or between sessions (SRD% = SRD/(mean of response) × 100). A value termed here as the minimum SRD range was also calculated. Each minimum SRD range was drawn around a reference line set to zero assuming no difference between two hypothetical measurements using the same approach (minimum SRD range = 0 ± SRD). Therefore, the minimum SRD range represents the minimum bounds for which we can expect to detect a true difference between two measurements using the same trial selection approach and criteria with 95% confidence^25^. The tighter the minimum SRD range, the more sensitive the method for enabling detection of difference between measurements not attributable solely to measurement error. Descriptive statistics summarizing trial and session characteristics are reported for valid trials from all subjects’ post data quality checks but prior to trial selection. All data and statistical analyses were performed using the R statistical computing software (R version 4.0.3, October 2020;^26^).

## Supplementary Information

Supplementary Information.

## Data Availability

Full datasets allowing for replication and reproduction of results, as well as the R code used here for analyses, are available upon request to the corresponding author.
