# Supplementary material for: An analysis of alternative forced oscillation technique reporting and validation methods for within- and between-sessions in healthy adults
Source: Sci Rep. 2022 Jul 30;12:13119. doi: 10.1038/s41598-022-17264-2 (PMC9338972; doi:10.1038/s41598-022-17264-2)
Supplement: Supplementary file 1 — Supplementary Information. [file 41598_2022_17264_MOESM1_ESM.pdf]

Supplemental Material

Title:
An analysis of alternative forced oscillation technique reporting and validation methods for within- and between-sessions in healthy adults.

Authors:
Jennifer H. Therkorn[1]
Wei Qian[1-2]
Daniella R. Toto[3]
*Michael J. Falvo[1,4]

Affiliations:

[1]Airborne Hazards and Burn Pits Center of Excellence, War Related Illness and Injury Study Center, VA New Jersey Heath Care System; East Orange, NJ

[2]Rutgers New Jersey Medical School, Rutgers Biomedical and Health Sciences; Newark, NJ

[3]School of Osteopathic Medicine, Rowan University; Stratford, NJ

[4]Departments of Pharmacology, Physiology & Neuroscience and Physical Medicine & Rehabilitation, Rutgers New Jersey Medical School; Newark, NJ

***Correspondence:**
Michael J. Falvo, PhD
VA NJ Health Care System
385 Tremont Ave
East Orange, NJ 07018
800-248-8005
Michael.Falvo@va.gov

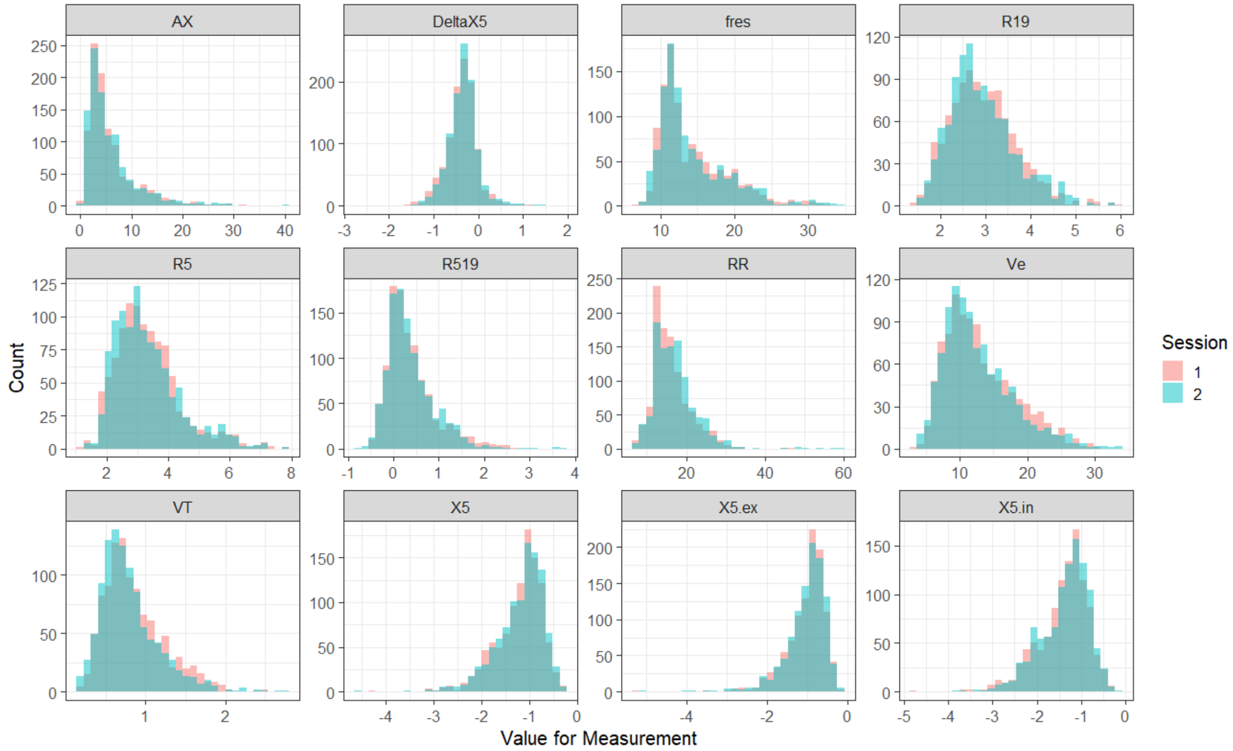

Figure S1. **Histograms (bin counts vs. measurement values) illustrating distributions for forced oscillation technique (FOT) variables measured in session 1 (red) and session 2 (blue).** FOT outcomes and units include the following: reactance area (AX; cm H_2O/L), whole breath low- (5 Hz) and mid-frequency (19 Hz) resistance and reactance (R5, R19, X5; cm H_2O ·s/L), low frequency reactance during inspiration and expiration (X5.in, X5.ex; cm H_2O ·s/L), frequency dependence of resistance (R519; cm H_2O ·s/L), DeltaX5 = X5.in – X5.ex [1]), resonant frequency (fres; Hz), respiratory rate (RR; breaths/min), minute ventilation (Ve; L/min), tidal volume (VT; L).

Table S1. **Summary statistics for forced oscillation technique (FOT) variables within and across sessions**. SD = variable's standard deviation; FOT outcomes and units include the following: reactance area (AX; cm H2O/L), whole breath low- (5 Hz) and mid-frequency (19 Hz) resistance and reactance (R5, R19, X5; cm H2O ·s/L), low frequency reactance during inspiration and expiration (X5.in, X5.ex; cm H2O ·s/L), frequency dependence of resistance (R519; cm H2O ·s/L), delta X5 = X5 inspiratory – X5 expiratory [1], resonant frequency (fres; Hz), respiratory rate (RR; breaths/min), minute ventilation (Ve; L/min), tidal volume (VT; L).

**Across Sessions**						
**Variable**	**n**	**Mean**	**SD**	**Median**	**Min**	**Max**
AX	2082	6.07	5.20	4.27	0.30	40.43
DeltaX5	2080	-0.36	0.35	-0.34	-2.72	1.88
fres	2081	14.43	4.77	12.53	7.07	34.74
R19	2082	2.96	0.73	2.85	1.44	5.98
R5	2082	3.34	1.06	3.14	1.23	7.89
R519	2082	0.38	0.54	0.25	-0.71	3.81
RR	2080	16.69	5.24	15.69	7.47	59.52
Ve	2080	12.91	5.14	11.74	3.45	33.79
VT	2080	0.83	0.37	0.74	0.14	2.72
X5	2082	-1.20	0.49	-1.09	-4.55	-0.26
X5.ex	2080	-1.03	0.49	-0.92	-5.25	-0.17
X5.in	2080	-1.39	0.56	-1.27	-4.86	-0.23
**Session 1**						
**Variable**	**n**	**Mean**	**SD**	**Median**	**Min**	**Max**
AX	1036	6.04	5.08	4.25	0.30	38.13
DeltaX5	1036	-0.38	0.35	-0.35	-2.72	1.11
fres	1036	14.43	4.68	12.50	7.07	32.38
R19	1036	2.97	0.73	2.89	1.44	5.98
R5	1036	3.35	1.06	3.17	1.23	7.84
R519	1036	0.38	0.54	0.24	-0.54	2.57
RR	1036	16.21	4.83	15.22	7.47	46.43
Ve	1036	13.10	5.15	11.96	3.45	29.97
VT	1036	0.86	0.38	0.77	0.16	2.50
X5	1036	-1.21	0.48	-1.10	-4.31	-0.29
X5.ex	1036	-1.03	0.48	-0.92	-5.25	-0.17
X5.in	1036	-1.41	0.57	-1.29	-4.86	-0.26
**Session 2**						
**Variable**	**n**	**Mean**	**SD**	**Median**	**Min**	**Max**
AX	1046	6.11	5.32	4.31	0.54	40.43
DeltaX5	1044	-0.34	0.35	-0.33	-1.58	1.88
fres	1045	14.42	4.87	12.56	7.65	34.74
R19	1046	2.94	0.73	2.81	1.56	5.79
R5	1046	3.33	1.06	3.10	1.36	7.89
R519	1046	0.38	0.54	0.25	-0.71	3.81
RR	1044	17.17	5.58	16.44	7.49	59.52
Ve	1044	12.72	5.12	11.46	4.01	33.79
VT	1044	0.79	0.37	0.72	0.14	2.72
X5	1046	-1.19	0.49	-1.07	-4.55	-0.26
X5.ex	1044	-1.03	0.50	-0.93	-5.16	-0.17
X5.in	1044	-1.37	0.54	-1.26	-3.77	-0.23

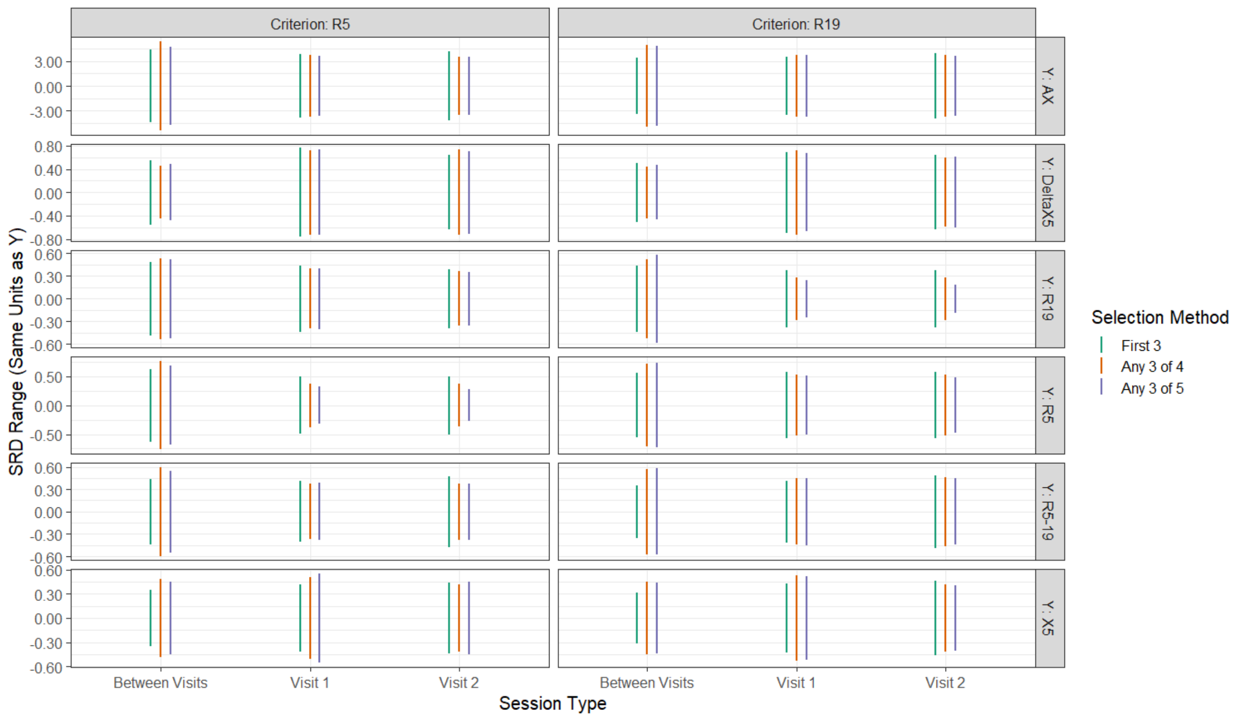

Figure S2. Smallest real differences as a function of criteria and selection methods within and between sessions. Minimum smallest real difference (SRD) range within and between sessions for each trial selection method stratified into two vertical sets of panels by coefficient of variation thresholding criteria and six horizontal sets of panels by outcome used as model fit response (Y's). This figure was created using data from all subjects able to achieve valid data while that in the main manuscript was created using the conservative subjects' dataset (able to achieve valid data across all selection methods and in both visits within each criteria). SRD units are the same as the outcomes: reactance area (AX, cm H_2O/L), all other outcomes (low- (5 Hz) and mid-frequency (19 Hz) resistance (R5, R19) and reactance (X5), frequency dependence of resistance (R5-19), delta X5 (Δ = X5 inspiratory – X5 expiratory;[1]), cm H_2O ·s/L). SRD was calculated as either the within or between session standard deviation (i.e., root mean square error from model fits) multiplied by √2x1.96 (SRD = SD x 2.77) [2]. What is termed here as the minimum SRD range was calculated as zero (i.e., assuming no difference between measurements using the same approach) ± SRD. Therefore, this SRD range represents the minimum difference required between two measurements using the same approach to conclude with 95% confidence the difference is not solely attributable to measurement error; therefore, smaller SRD ranges represent less measurement variability and greater sensitivity to detect a true difference [2,3].

Table S2. Within and between session variability across model fit response variables (Y's) for each trial selection method using R5 as the session validity thresholding criteria. This table was created using data from all subjects able to achieve valid data while that in the main manuscript was created using the conservative subjects' dataset (able to achieve valid data across all selection methods and in both visits within each criteria). R5, R19 and X5 = low- (5 Hz) and mid-frequency (19 Hz) resistance and reactance, respectively; R5-19 = frequency dependence of resistance; delta X5 = X5 inspiratory – X5 expiratory [1]) [all in units of cm H_2O ·s/L]; AX = reactance area [cm H_2O/L]; SD = standard deviation from model fit; SRD = smallest real difference = SD x 2.7 [2]; SRD% = SRD/(mean of response) x100); CoV = SD/(mean of response) x 100; * = variable spanning positive and negative values, so CoV is not calculated. Summary data for each variable, including response means used in calculations, can be found in the Supplementary Materials (Figure S1, Table S1).

Y	Session Type	Within Session 1			Within Session 2			Between Sessions		
	Selection Method	First 3	Any 3 of 4	Any 3 of 5	First 3	Any 3 of 4	Any 3 of 5	First 3	Any 3 of 4	Any 3 of 5
R5	SD	0.18	0.14	0.11	0.18	0.14	0.10	0.23	0.28	0.24
	SRD	0.49	0.38	0.31	0.50	0.37	0.27	0.63	0.76	0.68
	SRD%	0.14	0.11	0.09	0.15	0.11	0.08	0.19	0.23	0.20
	CoV	0.05	0.04	0.03	0.05	0.04	0.03	0.07	0.08	0.07
R19	SD	0.16	0.14	0.15	0.14	0.13	0.13	0.17	0.19	0.19
	SRD	0.43	0.40	0.41	0.39	0.36	0.35	0.48	0.53	0.53
	SRD%	0.15	0.13	0.14	0.13	0.12	0.12	0.16	0.18	0.18
	CoV	0.05	0.05	0.05	0.05	0.04	0.04	0.06	0.06	0.06
R5-19*	SD	0.15	0.13	0.14	0.17	0.14	0.14	0.16	0.21	0.20
	SRD	0.41	0.37	0.39	0.48	0.38	0.38	0.44	0.59	0.55
	SRD%	1.03	0.93	0.97	1.26	1.00	0.99	1.12	1.52	1.40
X5	SD	0.15	0.18	0.20	0.16	0.15	0.16	0.12	0.17	0.16
	SRD	0.42	0.51	0.55	0.44	0.42	0.45	0.34	0.48	0.45
	SRD%	0.35	0.42	0.45	0.37	0.35	0.38	0.29	0.40	0.38
	CoV	0.12	0.15	0.16	0.13	0.13	0.14	0.10	0.15	0.14
Delta X5*	SD	0.27	0.26	0.26	0.23	0.26	0.26	0.20	0.16	0.18
	SRD	0.76	0.72	0.73	0.64	0.73	0.71	0.55	0.45	0.49
	SRD%	2.03	1.94	1.95	1.90	2.16	2.10	1.56	1.27	1.37
AX	SD	1.40	1.35	1.30	1.50	1.27	1.27	1.58	1.93	1.70
	SRD	3.88	3.74	3.61	4.15	3.53	3.51	4.36	5.35	4.71
	SRD%	0.63	0.60	0.58	0.68	0.58	0.57	0.71	0.87	0.77
	CoV	0.23	0.22	0.21	0.25	0.21	0.21	0.26	0.31	0.28

References

1 Dellacà, R. L. *et al.* Detection of expiratory flow limitation in COPD using the forced oscillation technique. *European Respiratory Journal* **23**, 232-240, doi:10.1183/09031936.04.00046804 (2004).

2 Beckerman, H. *et al.* Smallest real difference, a link between reproducibility and responsiveness. *Qual Life Res* **10**, 571-578, doi:10.1023/a:1013138911638 (2001).

3 Bland, J. M. & Altman, D. G. Measurement error. *BMJ* **313**, 744, doi:10.1136/bmj.313.7059.744 (1996).
